# The interface between abiotic and biotic stress responses

**DOI:** 10.1093/jxb/erw110

**Published:** 2016-03-15

**Authors:** Walter Gassmann, Heidi M. Appel, Melvin J. Oliver

**Affiliations:** University of Missouri; University of Missouri; USDA - Agricultural Research Service

Organisms are under strong selection to respond adaptively to environmental stress, even when different stresses occur simultaneously or in rapid succession, as they often do in natural environments. However, at a molecular level, stress responses are often studied in isolation and under controlled growth conditions. This leaves us with an ever-finer picture of single stress responses but little understanding of how additional stressors modify those responses. Without companion studies of more complex systems of interacting stresses, we do not know how phenotypes are shaped under natural conditions. Based on whole organism studies in largely agricultural settings, we do know that stresses interact profoundly to shape phenotypes. We also have examples of cross-talk among signalling pathways associated with specific abiotic and biotic stress responses which hint at the existence of mechanisms that may integrate global plant stress responses. To move plant stress biology forward in transformative ways, we need greater collaboration among plant biologists studying different stresses in order to address the complexity of plant stress responses under natural conditions.

In 2015, the University of Missouri Interdisciplinary Plant Group (IPG) organized its annual symposium around the topic of *Plants Between a Rock and a Hard Place: The Interface between Plant Abiotic and Biotic Stress Responses*. A primary goal of the meeting was to promote and enhance multi-stress collaboration within the plant stress biology community by bringing together world-renowned experts in different aspects of plant stress biology who have already begun to study the interactions between different stresses. In this special issue of the *Journal of Experimental Botany*, we present some of the topics and research that were covered at the symposium.

Foyer *et al.* (2016) set the stage with a detailed look at how an abiotic stress can shape a plant’s response to attack by phloem-feeding aphids. They present a strong case to challenge the oft-held notion that abiotic stress events result in an increased susceptibility to biotic stress factors. The complex signalling pathways that direct plant responses to abiotic and biotic stressors overlap in many ways that lead to cross-tolerance phenomena. The plant’s response to aphid infestation involves interactions between hormone, redox, nitric oxide, kinase, and calcium signalling pathways that mirror aspects of the plant’s response to abiotic factors. The preponderance of evidence suggests that abiotic stressors do not predispose a plant to aphid attack and, in fact, may provide some measure of protection and that this may hold true for other abiotic–biotic stressor interactions.

Viruses depend on vectors such as plant-associated insects to move from plant to plant. To spread throughout an infected plant successfully and to facilitate vector transmission to another, viruses must manipulate the cellular processes of the host with a very limited set of proteins encoded in their small genomes. Studying these proteins from viruses and other plant-associated pathogens can provide novel insights into the processes involved in abiotic and biotic stress responses. Schoelz *et al.* (2016) provide an intriguing overview of the multifunctional P6 protein of *Cauliflower Mosaic Virus*, a novel function of which is to direct large protein assemblies devoted to virus replication into plasmodesmata for cell-to-cell movement.

In contrast to viruses, most microbial pathogens of plants do not enter the host cytoplasm. A first line of defence, therefore, occurs at the plant plasma membrane where receptors detect conserved non-self molecules collectively called microbe- or pathogen-associated molecular patterns (M/PAMPs) to initiate a broad defence response termed PAMP-triggered immunity (PTI) (Macho and Zipfel, 2014). While PAMPs and the corresponding plant receptors have been identified for a variety of microbial pathogens, a notable exception has been PAMPs derived from plant-parasitic nematodes. The review by Holbein *et al.* (2016) provides a fascinating update on progress in identifying elements of plant PTI responses to nematodes, thus broadening the arsenal for breeding resistance into crop plants.

Biotic and abiotic stressors often elicit systemic responses which depend on signalling from local sites of exposure to other areas of the plant. At the cellular or tissue level, the possible interplay between the various signalling pathways can be complex, especially when two or more stressors are combined as is often the case in the field. Huber and Bauerle (2016) describe the hydraulic, chemical, and electrical components that are part of the long-distance communication array of all plants. They provide a comprehensive assessment of what is known about how they operate and interact to co-ordinate a systemic response in the plant. The authors highlight the large gaps in knowledge that need to be addressed and make clear that combinations of stressors often elicit novel responses that differ from responses to single stressors.

Plant responses to abiotic and biotic stressors can lead to changes that appear to be ‘memorized’ and influence the stress tolerance of the next generation. The review by Bilichak and Kovalchuk (2016) explores possible epigenetic mechanisms associated with this phenomenon, highlighting the evidence that certain stress-induced epigenetic factors escape full-scale reprogramming of the epigenome during gametogenesis. The authors speculate that a better understanding of these epigenetic factors would enable the engineering of epigenetic modifications to cross-stress tolerance for crop improvement strategies in plant breeding.

The symposium was also an opportunity to showcase new and focused unpublished data. The research paper by MacQueen and Bergelson (2016) focuses on plant innate immunity to microbial pathogens. They show that abiotic factors such as temperature and humidity, which are known to influence pathogen aggressiveness, impact expression levels of plant resistance genes. These resistance genes provide a second line of defence against microbial pathogens, but their continued high expression reduces plant fitness. Therefore, modulation of resistance gene expression levels by abiotic factors probably reflects an adaptive advantage by balancing plant defence readiness and fitness in the absence of pathogens.

Finally, the research paper by Zhang *et al.* (2016) identifies an intriguing connection between the auxin and jasmonic acid (JA) signalling pathways via hormone metabolism. Even though auxin and JA regulate quite distinct developmental and stress response pathways, it was found that some of the indole-3-acetic acid (IAA) hydrolases (IAH) also metabolize JA-isoleucine, a bioactive derivative of JA. Manipulation of IAH expression levels led to surprising perturbations of these hormone signalling modules, highlighting the degree to which these two signalling pathways are interconnected via metabolic cross-talk.

Worldwide, humans rely on plants for food, fibre, and fuel, and face the daunting task of growing food for nine billion people by 2050 while reducing the carbon, fertilizer, and water footprint of agriculture. In addition, the consensus prediction is that global climate change will destabilize plant growth conditions in terms of higher CO_2_ levels and increased risks of severe fluctuations in temperature and precipitation (Schroeder *et al.*, 2013). These adverse abiotic conditions will most likely favour the spread of plant pathogens and pests into new geographic areas. Such a scenario increases the need to develop abiotic stress and pathogen- and pest-resistant crop plants at a speed that cannot be met by breeding alone (Dangl *et al.*, 2013). The combination of abiotic and biotic stresses not only threatens agriculture but also plants in natural environments that fulfil important ecosystem services. Addressing these major challenges will require an interdisciplinary and concerted approach. We hope that our readers will find, as we did, that the reviews and research articles in this special issue provide a useful and stimulating contribution towards addressing these challenges.

We are grateful to the sponsors of our symposium, in particular the National Science Foundation, the USDA National Institute of Food and Agriculture, and the *Journal of Experimental Botany* for their support.
